# Excess natural-cause deaths in California by cause and setting: March 2020 through February 2021

**DOI:** 10.1093/pnasnexus/pgac079

**Published:** 2022-06-08

**Authors:** Yea-Hung Chen, Andrew C Stokes, Hélène E Aschmann, Ruijia Chen, Shelley DeVost, Mathew V Kiang, Suneil Koliwad, Alicia R Riley, M Maria Glymour, Kirsten Bibbins-Domingo

**Affiliations:** Department of Epidemiology and Biostatistics, University of California, 550 16th St, San Francisco, CA 94158, USA; Department of Global Health, Boston University School of Public Health, Boston, MA 02118, USA; Department of Epidemiology and Biostatistics, University of California, 550 16th St, San Francisco, CA 94158, USA; Department of Epidemiology and Biostatistics, University of California, 550 16th St, San Francisco, CA 94158, USA; Department of Epidemiology and Biostatistics, University of California, 550 16th St, San Francisco, CA 94158, USA; Department of Epidemiology and Population Health, Stanford University School of Medicine, Palo Alto, CA 94304, USA; Department of Medicine, and Diabetes Center, University of California, San Francisco, CA 94117, USA; Department of Sociology, University of California, Santa Cruz, CA 95064, USA; Department of Epidemiology and Biostatistics, University of California, 550 16th St, San Francisco, CA 94158, USA; Department of Epidemiology and Biostatistics, University of California, 550 16th St, San Francisco, CA 94158, USA

**Keywords:** Covid-19, excess mortality, cause of death, home deaths

## Abstract

Excess mortality has exceeded reported deaths from Covid-19 during the pandemic. This gap may be attributable to deaths that occurred among individuals with undiagnosed Covid-19 infections or indirect consequences of the pandemic response such as interruptions in medical care; distinguishing these possibilities has implications for public health responses. In the present study, we examined patterns of excess mortality over time and by setting (in-hospital or out-of-hospital) and cause of death using death certificate data from California. The estimated number of excess natural-cause deaths from 2020 March 1 to 2021 February 28 (69,182) exceeded the number of Covid-19 diagnosed deaths (53,667) by 29%. Nearly half, 47.4% (32,775), of excess natural-cause deaths occurred out of the hospital, where only 28.6% (9,366) of excess mortality was attributed to Covid-19. Over time, increases or decreases in excess natural non-Covid-19 mortality closely mirrored increases or decreases in Covid-19 mortality. The time series were positively correlated in out-of-hospital settings, particularly at time lags when excess natural-cause deaths preceded reported Covid-19 deaths; for example, when comparing Covid-19 deaths to excess natural-cause deaths in the week prior, the correlation was 0.73. The strong temporal association of reported Covid-19 deaths with excess out-of-hospital deaths from other reported natural-cause causes suggests Covid-19 deaths were undercounted during the first year of the pandemic.

Significance Statement Prior studies have established that excess mortality exceeded Covid-19 mortality during the first year of the pandemic. This study offers evidence that excess deaths not assigned to Covid-19 primarily represent undiagnosed Covid-19 deaths, rather than deaths indirectly related to the pandemic. Undercounting of Covid-19 deaths, which is more prevalent for out-of-hospital deaths, points to significant deficiencies in the death investigation system. These inaccuracies may reduce the ability to prepare for and respond to the Covid-19 pandemic effectively, leading to incorrect prioritization of public health strategies. As the pandemic continues, efforts to reform the death investigation system and improve cause-of-death reporting are urgently needed, such as increasing resources to death certifiers, improving training, and eliminating archaic structures such as the sheriff–coroner system.

## Introduction

The estimated number of excess deaths occurring during the Covid-19 pandemic—the observed number of deaths minus the number of the deaths expected had the pandemic not occurred—exceeds the reported number of Covid-19 deaths in the United States (1, 2). For example, Woolf and colleagues estimated a total of 522,368 (95% CI: 521,413 to 523,322) excess deaths of any cause in the United States from March through December 2020, of which only 378,039 deaths were attributed to Covid-19 (2). The discrepancy has at least three possible explanations. (i) It may reflect under-reporting of Covid-19 as a cause of death (3, 4). This may have occurred due to the absence of widespread testing in the community, certifiers’ lack of familiarity with the clinical manifestations of the disease, or under-resourced death-investigation systems (5). (ii) The pandemic may have increased mortality via mechanisms other than Covid-19, such as decreases in healthcare utilization, reduced social support, or economic hardship (6–10). (iii) Individuals who have recovered from Covid-19 may have increased risks for mortality, via mechanisms not involving acute Covid-19 (11, 12). Evaluating these theories is important for understanding the full impact of Covid-19 and prioritizing public health responses.

Pandemic-related increases in causes of death other than Covid-19 have been reported elsewhere (13–15). A review of US data found 15% more deaths due to diabetes in 2020 than in 2019, and 10% and 6% more deaths due to Alzheimer's disease and stroke, respectively (13). Similarly, an analysis found 11% more deaths per week in the United States due to ischemic heart disease in 2020 versus in 2019 (14).

California, with substantial geographic and racial/ethnic diversity and nearly 40 million residents, is an ideal setting to evaluate why deaths from causes other than Covid-19 increased during the pandemic. Using decedent-level death data from the California Department of Public Health, we estimated excess mortality by setting of death (whether in-hospital or out-of-hospital) and cause of death to examine two testable hypotheses.

First, we reasoned that the *discrepancy* between excess natural-cause deaths and Covid-19 deaths would vary across setting of death: whether in-hospital or out-of-hospital. Because California hospitals initiated routine Covid-19 testing of inpatients soon after the start of the pandemic (as mandated by California's Department of State Hospitals), in-hospital deaths from undiagnosed Covid-19 in the state are likely rare. Any in-hospital (positive or negative) excess deaths from natural causes other than Covid-19 should, therefore, reflect true excess. We, thus reasoned that in-hospital excess non-Covid-19 mortality would be low, and possibly further minimized by shifts in setting of death due to hospital aversion. Conversely, we hypothesized that in out-of-hospital settings, excess non-Covid-19 mortality would be high, reflecting either undiagnosed infections attributed to another cause—particularly because postmortem testing for those who died out of the hospital has been documented to be inconsistent (5)—or shifts in setting of death due to hospital aversion.

Second, we theorized that *temporal trends* in excess natural-cause deaths, and correlations between excess natural-cause deaths and reported Covid-19 deaths, would vary by setting of death. Specifically, in out-of-hospital settings, we theorized that increases in excess natural-cause deaths might slightly precede increases in diagnosed Covid-19 deaths. We reasoned that peaks in deaths due to undiagnosed Covid-19 are likely to occur earlier than peaks in deaths due to diagnosed Covid-19, because case recognition likely improves as a surge progresses, diagnosis may extend life, and each additional day of having a life-threatening infection increases the chances a person seeks care and receives a diagnosis. In in-hospital settings, we hypothesized that peaks in excess natural-cause deaths would occur at the same time or slightly after peaks in deaths from diagnosed Covid-19. We reasoned here that the burden on the healthcare system might cause delays in care, leading to declines in in-hospital deaths.

## Results

**Fig. 1.**
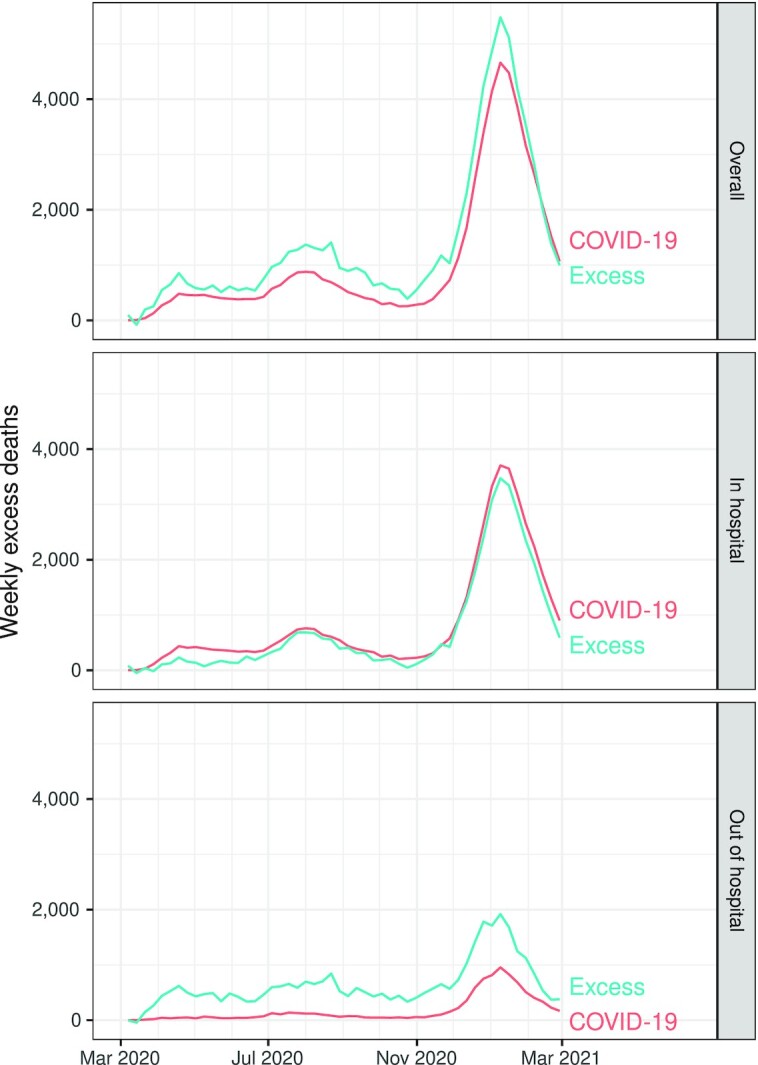
Weekly excess natural-cause natural deaths and Covid-19 deaths, California, March 2020 through February 2021.

Figure 1 shows excess natural-cause deaths and reported Covid-19 deaths by week, by setting of death. In both settings (in-hospital and out-of-hospital), there was (positive) excess mortality across the entire time, peaking in January 2021. Overall (across settings), there were more excess deaths than reported Covid-19 deaths. This pattern differed across setting of death: in in-hospital settings there were more reported Covid-19 deaths than excess deaths, whereas in out-of-hospital settings there were far more excess deaths than reported Covid-19 deaths. Temporal correspondence between peaks in Covid-19 deaths and excess deaths was observed in in-hospital settings and in out-of-hospital settings, as well as overall across both settings.

Table 1 shows excess deaths during the first year of the pandemic, by setting and cause of death, for all natural-cause deaths in California not occurring in nursing homes or hospices. In in-hospital settings, there were 1.47 (95% Prediction Interval [PI] 1.44 to 1.50) times more deaths than expected according to prepandemic deaths, while in out-of-hospital settings there were 1.26 (95% PI 1.23 to 1.30) times more deaths than expected. The estimated number of excess natural-cause deaths (69,182) exceeded the number of diagnosed Covid-19 deaths (53,667), implying that diagnosed Covid-19 deaths accounted for 78% of excess natural-cause deaths. We estimated 36,407 (95% PI: 34,718 to 38,096) excess natural-cause deaths in in-hospital settings, 18% less than the reported number of in-hospital Covid-19 deaths (44,301). For several causes of death, including Alzheimer's disease and related dementias (ADRD), cardiovascular diseases, cerebrovascular diseases, malignant neoplasms, and other respiratory diseases, the number of in-hospital deaths that occurred was less than expected. For example, we estimated 22% (RR: 0.78, 95% PI: 0.75 to 0.81) or 3,652 (95% PI: 2,968 to 4,331) fewer deaths from malignant neoplasms in in-hospital settings than expected.

**Table 1.** Excess mortality among Californians by place of death and cause of death, excluding deaths occurring in nursing facilities or hospices, March 2020 through February 2021.

	In-hospital deaths		Out-of-hospital deaths		
	Excess deaths	Risk ratio	Excess deaths	Risk ratio	Net (%*)
Natural causes	36,407 (34,718 to 38,096)	1.47 (1.44 to 1.50)	32,775 (29,509 to 36,082)	1.26 (1.23 to 1.30)	69,182
ADRD^†^	−346 (−624 to −69)	0.86 (0.78 to 0.97)	2,587 (1,657 to 3,515)	1.19 (1.11 to 1.27)	2,240 (14.4)
Cardiovascular diseases	−1,930 (−2,520 to −1,336)	0.90 (0.87 to 0.93)	7,649 (5,762 to 9,539)	1.19 (1.14 to 1.25)	5,719 (36.9)
Cerebrovascular diseases	−516 (−658 to −373)	0.92 (0.90 to 0.94)	1,982 (1,450 to 2,515)	1.32 (1.22 to 1.45)	1,467 (9.5)
Certain infectious and parasitic diseases	−134 (−404 to 139)	0.96 (0.89 to 1.05)	178 (59 to 298)	1.23 (1.07 to 1.45)	45 (0.3)
COVID-19	44,301		9,366		53,667
Diabetes mellitus	51 (−143 to 247)	1.02 (0.95 to 1.09)	2,050 (1,661 to 2,443)	1.37 (1.28 to 1.48)	2,101 (13.5)
Digestive diseases	1 (−643 to 650)	1.00 (0.91 to 1.10)	1,023 (955 to 1,092)	1.32 (1.29 to 1.35)	1,024 (6.6)
Endocrine, nutritional, and metabolic diseases	−22 (−203 to 160)	0.98 (0.85 to 1.16)	919 (688 to 1,152)	1.41 (1.28 to 1.57)	897 (5.8)
Genitourinary diseases	89 (−164 to 346)	1.03 (0.95 to 1.12)	472 (289 to 658)	1.25 (1.14 to 1.39)	562 (3.6)
Influenza and pneumonia	−626 (−1,324 to 72)	0.87 (0.76 to 1.02)	−43 (−233 to 146)	0.95 (0.78 to 1.22)	−669
Malignant neoplasms	−3,652 (−4,331 to −2,968)	0.78 (0.75 to 0.81)	5,536 (5,090 to 5,981)	1.16 (1.15 to 1.18)	1,883 (12.1)
Mental and behavioral disorders	−15 (−89 to 60)	0.98 (0.87 to 1.11)	464 (431 to 497)	1.50 (1.45 to 1.56)	449 (2.9)
Other diseases of the nervous system	−141 (−336 to 57)	0.92 (0.83 to 1.04)	678 (253 to 1,107)	1.19 (1.06 to 1.36)	537 (3.5)
Other respiratory diseases	−1,474 (−1,875 to −1,069)	0.78 (0.74 to 0.83)	760 (475 to 1,045)	1.09 (1.06 to 1.13)	−714
Other natural causes	−285 (−378 to −193)	0.92 (0.89 to 0.94)	252 (184 to 320)	1.14 (1.10 to 1.19)	−33
Decomposition discrepancy^‡^	1106		−1,098		8
Non-COVID-19 natural causes	−7,894 (−9,583 to 20,143)	0.90 (0.88 to 1.16)	23,409 (20,143 to 20,143)	1.19 (1.16 to 1.16)	15,515

* The percent of non-COVID-19 excess mortality (last row).

† Alzheimer's disease and related dementias.

‡ The estimated number of excess deaths (first row) minus the sum across specific causes of deaths (all other rows excluding the last one). The discrepancy occurs because each cause of death is modeled separately.

There were 9,366 Covid-19 deaths reported in out-of-hospital settings; this represents 18% of all reported Covid-19 deaths and 29% of excess natural-cause deaths in out-of-hospital settings. We estimated excess out-of-hospital deaths for several specific causes. For example, we estimated 16% (RR: 1.16; 95% PI: 1.15 to 1.18) or 5,536 (95% PI: 5,090 to 5,981) more out-of-hospital deaths from malignant neoplasms occurred than we would have expected based on prepandemic trends. In many cases, the number of in-hospital deaths was lower than expected, but in-hospital declines (relative to the counterfactual) were more than offset by elevated out-of-hospital deaths, such that total cause-specific mortality was higher than expected during the pandemic. For example, for malignant neoplasms, the negative excess of 3,652 in in-hospital settings was offset by the positive excess of 5,536 in out-of-hospital settings, resulting in a net of 1,883. In total, across settings, there were 15,515 excess natural-cause deaths not attributed to Covid-19. Cardiovascular diseases accounted for the largest proportion of this excess (36.9%), followed by diabetes (13.5%) and malignant neoplasms (12.1%).

**Fig. 2.**
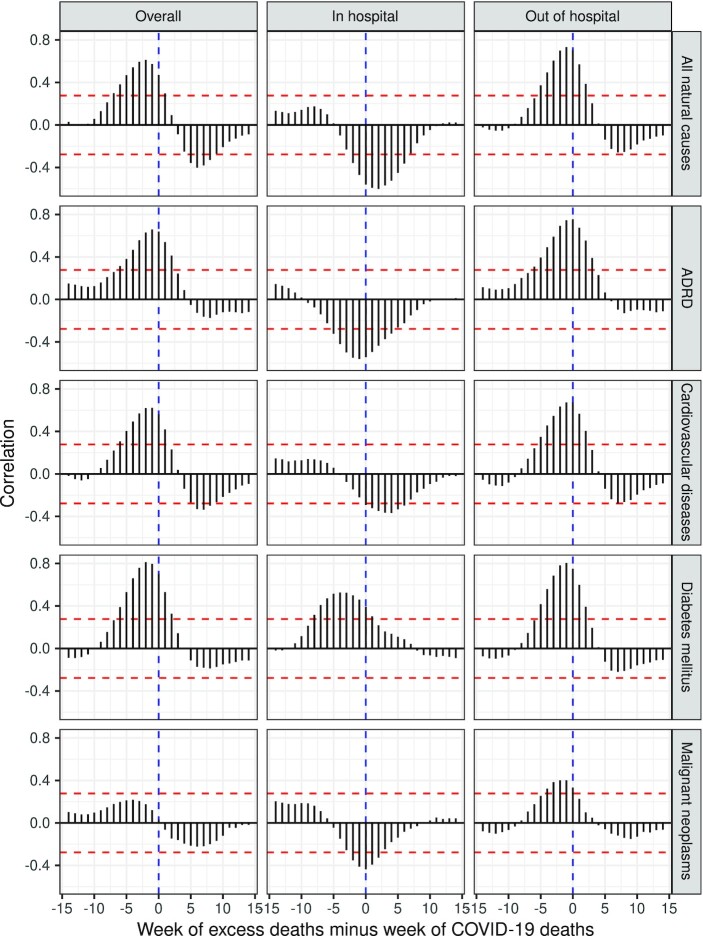
Correlograms of excess natural-cause deaths (excluding Covid-19) and Covid-19 deaths, by setting of death and cause of death, California, March 2020 through February 2021. The *x-*axis is the time lag of excess deaths relative to Covid-19 deaths. The time lag is 0 when the weeks are aligned; negative when comparing Covid-19 deaths to excess non-Covid-19 deaths in prior weeks; and positive when comparing Covid-19 to excess non-Covid-19 deaths in subsequent weeks. Dashed red lines indicate significance of the correlation coefficients at alpha = 0.05. ADRD refers to Alzheimer's disease and related dementias.

Figure 2 shows temporal correspondence between weekly Covid-19 deaths and weekly excess natural-cause deaths (excluding Covid-19) across varying lag periods, by cause and setting of death. Overall (across setting), the correlation was positive at a lag of 0 (*r* = 0.47), and statistically significant and positive at lags of −7 (when comparing Covid-19 deaths to excess deaths occurring 7 weeks prior) through 1 (when comparing Covid-19 deaths to excess deaths occurring 1 week later). The largest positive correlation occurred at a lag of −2 weeks (*r* = 0.61). These patterns varied by setting of death. In in-hospital settings, the correlation was negative at a lag of 0 (*r* = −0.56), statistically significant and negative between lags of −2 through 6 weeks, and largest in magnitude at a lag of 2 weeks (*r* = −0.60). In comparison, in out-of-hospital settings, the correlation was positive at a lag of 0 (*r* = 0.71), statistically significant and positive between lags of −5 through 2, and largest in magnitude at a lag of −1 week (*r* = 0.73).

Similar patterns were apparent for several specific causes of death. For example, for deaths due to malignant neoplasms, the correlation at lag 0 was negative in in-hospital settings (*r* = −0.44) and positive in out-of-hospitals settings (*r* = 0.33). In-hospital deaths from diabetes, however, were positively associated with in-hospital Covid-19 deaths at a lag of 0 (*r* = 0.39), peaking in magnitude at a lag of −4 (*r* = 0.53).

## Discussion

As with prior national analyses, excess mortality in California during the first year of the pandemic exceeded diagnosed Covid-19 deaths. In our analysis limited to natural causes of death, this discrepancy is estimated at 29%. The four lines of evidence suggest that a large fraction of the discrepancy is due to undiagnosed Covid-19.

First, consistent with our first hypothesis, the estimated number of excess deaths far exceeded the reported number of Covid-19 deaths in out-of-hospital settings, where testing for Covid-19 is known to be incomplete. In fact, we found that excess non-Covid-19 natural-cause mortality only occurred in out-of-hospital settings, confirming that factors which differ by setting of death are key to explaining the discrepancy between excess natural-cause and Covid-19 mortality.

Second, we found temporal correspondence between excess non-Covid-19 deaths and reported Covid-19 deaths, as predicted in our second hypothesis. In out-of-hospital settings, increases in excess deaths emerged most strongly at 2 to 3 weeks prior to peak deaths due to diagnosed Covid-19. A combination of processes may contribute to an early peak in deaths due to undiagnosed Covid-19. At the beginning of a surge, it may be less likely that a case is recognized as Covid-19, so deaths from undiagnosed Covid would naturally precede deaths from diagnosed cases. Diagnosis increases the likelihood of treatment, and may thereby extend life. Finally, naturally occurring heterogeneity in the clinical course of disease, combined with an increase in chances of diagnosis with each additional day after infection, would create an earlier peak in deaths due to undiagnosed Covid-19. If the Covid-19 pandemic response had indirectly increased mortality due to delays in medical care or general instability, we would expect such non-Covid-19 deaths to peak during and in the weeks *following* peak reported Covid-19 mortality. Similarly, such a pattern would be expected if excess mortality was occurring among individuals previously infected with SARS-CoV-2. To the contrary, overall non-Covid-19 excess deaths dip in the weeks following increases in Covid-19 mortality. The positive association between Covid-19 deaths and excess non-Covid-19 deaths in out-of-hospital settings also suggests underreporting of Covid-19 deaths, since both diagnosed and undiagnosed Covid-19 will be influenced by the community prevalence of disease.

Third, among out-of-hospital deaths, a strong positive correlation was identified for causes of death which are unlikely to be immediately fatal due to delayed medical care, such as ADRD and cancer. Although these conditions can lead to death, and high-quality medical care might delay death, increases in ADRD or cancer mortality from delayed care should accumulate slowly over time. The fact that deaths from these causes increased rapidly in tandem with Covid-19 deaths suggests that most of the excess deaths from ADRD and cancer in out-of-hospital settings are instead likely due to undiagnosed Covid-19.

The fourth line of evidence in support of the undiagnosed Covid-19 explanation is that some of the largest relative excess mortality in out-of-hospital settings occurred for causes of death known to sometimes result from SARS-CoV-2 infection (such as cerebrovascular disease) or to substantially elevate the mortality risk for Covid-19 patients (such as diabetes). It is possible that some deaths may not have been classified as involving Covid-19 due to the absence of acute disease, but that Covid-19 was still an underlying cause of death and should have been listed in the death records.

The setting-stratified analysis also suggests displacement of deaths from in-hospital settings to out-of-hospital settings. In in-hospital settings, Covid-19 deaths were negatively associated with excess non-Covid-19 natural-cause deaths in subsequent weeks, consistent with decreased hospital utilization during Covid-19 surges. We speculate that hospital utilization declined in the wake of reports that Covid-19 case numbers were high. Indeed, several studies have documented declines in hospital utilization, particularly early in the pandemic (7–9). However, the excess deaths in out-of-hospital settings far exceeded the decline in in-hospital settings; the net of these in the community-dwelling population is, as explained above, largely attributable to Covid-19 (whether diagnosed or undiagnosed).

In hospital settings, the patterns of correlation for diabetes and ADRD mortality differed from the patterns observed for other causes of death. For example, excess diabetes mortality was positively associated with COVID-19 mortality in subsequent weeks in hospital settings. This result suggests that in hospital settings, some COVID-19 deaths were misdiagnosed as diabetes deaths. This could have occurred due to the complexity of assigning cause of death when diabetes is involved. ADRD mortality was associated with lower reported COVID-19 mortality in subsequent weeks in hospital settings. This finding could reflect frailty selection if early deaths from undiagnosed COVID-19 offset later deaths from diagnosed COVID-19 in hospital settings.

Our interpretation that the discrepancy between Covid-19 deaths and excess deaths is primarily due to undiagnosed Covid-19 deaths in out-of-hospital settings does not rule out other explanations. The pattern of weekly correlations in out-of-hospital settings—with peak correlations observed for excess deaths occurring 2 to 3 weeks prior to reported Covid-19 deaths—could reflect deaths associated with increased hospital strain in the weeks prior to peak reported Covid-19 mortality. However, we would expect that if this were the primary explanation for the finding, the time lag of the maximum correlation would be zero or positive rather than negative. That is, delays in accessing urgent medical care should be most pronounced in the weeks concurrent with or following peak reported COVID-19 deaths—when hospital capacity is most constrained—rather than in the weeks preceding peak reported COVID-19 deaths—when hospital capacity remains less constrained. Nonetheless, several recent studies suggest that hospital strain may have contributed to increases in mortality during the pandemic. One recent study, for example, estimated that an upper bound of 7.9% of excess veteran deaths early in the pandemic may have been associated with hospital avoidance (7). Similarly, a study of cardiovascular deaths during the pandemic (14) concluded that the pandemic “may have had an indirect toll on patients.” Such an effect, if true, may concentrate among specific diseases that are especially sensitive to healthcare access. In particular, increases in deaths related to diabetes (representing 14% of excess non-Covid-19 natural-cause mortality in our analysis) most plausibly reflect the complexity of care and the impact that deteriorations in the care network would have had on the level of support for fragile patients. For example, prescriptions for insulin filled in US retail pharmacies declined at the onset of the pandemic and through 2020 (16). Moreover, excess diabetes deaths have been reported after natural disasters (17), further supporting the hypothesis that environmental stressors can quickly disrupt self-care and support systems and increase mortality (18). Nonetheless, diabetes substantially increases mortality among Covid-19 patients and would, therefore, also likely be overrepresented in fatalities due to undiagnosed Covid-19.

We observed negative excess mortality net of Covid-19 in hospital settings, which we interpreted as evidence of shifts in the setting of death due to hospital aversion. However, as with out-of-hospital deaths, this interpretation does not rule out other potential explanations. Another potential factor that may have contributed to lower than expected natural-cause deaths in hospital settings during the pandemic is frailty selection. This could have occurred if those who died from Covid-19 were exceptionally frail and would have otherwise died of another condition, such as cardiovascular disease or cancer, in the absence of the pandemic. For influenza and pneumonia and other respiratory diseases specifically, lower than expected mortality levels in hospital settings during the pandemic were likely driven at least in part by genuine reductions in mortality from these conditions due to Covid-19 mitigation efforts, such as shelter-in-place, masking, and social distancing measures.

Our study underscores the likelihood of inaccuracies in reporting of cause of death in the United States, in the context of the Covid-19 pandemic. Challenges in reporting cause of death have been previously documented, including during the pandemic (19–22). Similarly, a recent study found discrepancies between the true prevalence of prior SARS-CoV-2 infection and reported case counts, pointing to insufficient access to testing (23). Inadequate Covid-19 testing in community settings may have contributed to inaccurate counts of Covid-19 deaths in out-of-hospital settings. The low quality of cause-of-death reporting for out-of-hospital deaths may also relate to characteristics of the death investigation system (24). In California, some death investigations, especially in out-of-hospital settings, are performed by county coroners who are elected officials with limited medical training and serve in a dual capacity as sheriff–coroner. Inaccuracies in cause of death reporting, which appear to primarily occur in out-of-hospital settings, reduce the ability to prepare for and respond to the Covid-19 pandemic. Data on Covid-19 mortality are used in numerous policy decisions, mathematical forecasts for disease trajectory, and estimates of case fatality rates. Incomplete identification of Covid-19 deaths has likely led to underestimation of the consequences of the pandemic, and incorrect prioritization of possible public health prevention strategies. Prior work has identified especially severe discrepancies between Covid-19 deaths and excess deaths in US counties with low socioeconomic status, a greater proportion of Black people, and higher prevalence of comorbidities (25).

Further studies will be needed to more thoroughly understand discrepancies between excess deaths and Covid-19 deaths. We recommend, in particular, reviewing medical charts or including interviews with next-of-kin, which could help ascertain consistency between recorded death-certificate data and other sources. Examining the demographic characteristics of excess deaths by setting of death was outside the scope of the present study, but represents another important direction for future research. Specifically, understanding whether out-of-hospital excess deaths have disproportionately occurred among older adults or among communities of color may help clarify the underlying cause of the increases and inform public health policies to reduce out-of-hospital deaths in the future. Future research should also evaluate the generalizability of the findings to other geographic locations. In particular, underreporting of Covid-19 deaths in out-of-hospital settings may differ across state contexts depending on levels of community testing, the quality and availability of healthcare, and characteristics of the death investigation system. For example, more resources and capacity for postmortem testing in states with a centralized medical examiner system may lead to fewer uncounted Covid-19 deaths in out-of-hospital settings.

In conclusion, our analyses add insight to understanding estimates of excess mortality associated with the Covid-19 pandemic. Several features of the association between Covid-19 deaths and excess deaths attributed on death certificates to natural causes other than Covid-19 suggest many of these deaths may have been due to undiagnosed Covid-19. This suggests that the death toll due to Covid-19 may have been substantially larger than previously reported.

## Materials and Methods

We obtained individual-level data from the California Department of Public Health on all deaths occurring on or after 2016 January 1. Our study was approved by the State of California Committee for the Protection of Human Subjects. We restricted analysis to California residents who died outside of a nursing facility or hospice, in order to focus on the community-dwelling California population (under the rationale that community-level trends in Covid-19 deaths were less likely to be related to trends among confined dwellers). We additionally restricted to natural-cause deaths. Our time window of interest was 2020 March 1 through 2021 February 28.

Our outcomes were defined by specific cause of death and setting of death as recorded on death certificates. We classified setting of death as in-hospital or out-of-hospital, and included dead-on-arrival and emergency-department deaths in the out-of-hospital category, as these deaths occur among individuals who were not in-hospital patients immediately before death. We include 15 natural causes: ADRD; cardiovascular diseases; cerebrovascular diseases; certain infectious and parasitic diseases; Covid-19; diabetes mellitus; digestive diseases; endocrine; nutritional and metabolic diseases; genitourinary diseases; influenza and pneumonia; malignant neoplasms; mental and behavioral disorders; other behaviors of the nervous system; other respiratory diseases; and other natural causes. We identified these causes using ICD-10 codes from the field for the underlying (i.e. final) cause of death. We defined Covid-19 deaths using all 20 cause-of-death fields available from the death certificate; this definition overwrites any other underlying cause of death. We chose to prioritize Covid-19 as a cause of death because we wanted to parse out deaths that were in no way attributed to Covid-19 even when such attribution should have occurred. To accomplish this, we only assigned deaths to other natural causes if there was no mention of Covid-19 anywhere on the death certificate, thus ensuring that all causes of death included in the analysis were mutually exclusive. However, in over 95% of the cases when Covid-19 was listed as a cause of death, it was listed as the primary cause of death. ICD-10 codes for our 15 natural causes are included in Table S1 (Supplementary Material).

We estimated excess mortality for each cause and setting of death using time-series analyses to account for historical mortality trends and seasonality. Our methods are described in detail in prior manuscripts (26, 27), but briefly, for each group of interest, we repeated the following procedure. We aggregated the data to weeks or months, using monthly data for estimates of cumulative excess mortality and weekly data for estimates of weekly excess mortality. We then fit dynamic harmonic regression models with autoregressive integrated moving average (ARIMA) errors for the number of weekly or monthly all-cause deaths, using deaths occurring among the group between 2016 January 1 and 2020 February 29. For each iteration, we used a model-fitting procedure described by Hyndman and Khandakar (28). Using the final model, we forecast the number of deaths for each unit of time, along with corresponding 95% PI.

To obtain the total number of excess deaths for the entire time window (2020 March 1 through 2021 February 28), we subtracted the total number of expected (forecast) deaths from the total number of observed deaths. To obtain relative excess mortality, we divided the observed number of deaths by the expected number of deaths. The relative excess mortality is interpretable as the risk ratio for mortality, comparing the pandemic to the counterfactual of no pandemic. We obtained 95% PI for expected mortality by simulating the model 10,000 times and selecting the 2.5% and 97.5%. We used these to obtain prediction intervals for both measures of excess mortality—number of excess deaths and relative excess mortality—by subtraction or division. As we modeled each cause of death separately, we also calculated a decomposition discrepancy reflecting the difference between estimated natural-cause excess deaths and the sum of estimates for each specific cause of death.

To assess the temporal association between reported Covid-19 deaths and excess deaths not attributed to Covid-19, we used correlograms, a visual tool plotting time lag on the *x-*axis and correlation on the *y-*axis. Under this strategy, we treated the weekly Covid-19 and excess non-Covid-19 deaths as two time series. We plotted correlations between values of the two time series at various time lags, including: when the weeks are aligned (a lag of 0), when comparing Covid-19 deaths to excess non-Covid-19 deaths in prior weeks (negative lags), and when comparing Covid-19 to excess non-Covid-19 deaths in subsequent weeks (positive lags). We produced correlograms overall and stratified by cause and by setting of death. These plots facilitated examination of the correlation between Covid-19 deaths and excess deaths at different time lags and in different settings. We conducted all analyses in R, version 4.04.

## Supplementary Material

pgac079_Supplemental_File

## Data Availability

The data are owned by the Vital Statistics, California Department of Public Health. Other parties can apply with the California Department of Public Health to receive that data. A contact email address for this process is: cphs-mail@oshpd.ca.gov.
