# Supplementary material for: Excess natural-cause deaths in California by cause and setting: March 2020 through February 2021
Source: PNAS Nexus. 2022 Jun 8;1(3):pgac079. doi: 10.1093/pnasnexus/pgac079 (PMC9272175; doi:10.1093/pnasnexus/pgac079)
Supplement: pgac079_Supplemental_File [file pgac079_supplemental_file.docx]

Table S1: Causes of death and corresponding ICD-10 codes.

| **Causes of death** | **ICD-10 codes** |
| --- | --- |
| Alzheimer’s disease and related dementias | F01-F04, G30, G31 |
| Cardiovascular diseases | I, excluding I60-I69 |
| Cerebrovascular diseases | I60-I69 |
| Certain infectious and parasitic diseases | A, B |
| COVID-19 | U07 |
| Diabetes mellitus | E10-E14 |
| Digestive diseases | K |
| Endocrine, nutritional, and metabolic diseases | E, excluding E10-E14 |
| Genitourinary diseases | N |
| Influenza and pneumonia | J10-J18 |
| Malignant neoplasms | C |
| Mental and behavioral disorders | F, excluding F01-F04 |
| Other diseases of the nervous system | G, excluding G30 and G31 |
| Other respiratory diseases | J, excluding J10-J18 |
